# Characteristics of cervical sagittal parameters in healthy cervical spine adults and patients with cervical disc degeneration

**DOI:** 10.1186/s12891-018-1951-8

**Published:** 2018-02-02

**Authors:** Rong Xing, Wangmi Liu, Xilei Li, Libo Jiang, Mumingjiang Yishakea, Jian Dong

**Affiliations:** Department of Orthopaedic Surgery, Zhongshan Hospital, Fudan University, 180 Fenglin Road, Shanghai, 200032 China

**Keywords:** T1 slope, Cervical disc degeneration, Cervical sagittal alignment, Radiograph, Cervical sagittal parameter

## Abstract

**Background:**

The cervical sagittal parameters of the normal population and the impact of disc degeneration on cervical sagittal alignment have not been clearly defined yet. This study is applied to investigate the characteristics and relationships of cervical sagittal parameters in normal adults and patients with cervical disc degeneration.

**Methods:**

We reviewed 50 normal control subjects (normal group, NG) and 50 patients with cervical disc degeneration (degeneration group, DG), who had both cervical MRI and radiographs obtained together, between January 2010 and September 2015. Data including C2–7 lordosis (CL), T1 slope (T1S), thoracic inlet angle (TIA), neck tilt (NT), C2–7 sagittal vertical axis (C2–7 SVA), cervical tilting, and cranial tilting on cervical radiographs were collected and analyzed.

**Results:**

T1S in the NG was significantly greater than in the DG (*P* < 0.05), while NT and C2–7 SVA in the NG were significantly lower than in the DG (*P* < 0.01 and *P* < 0.05, respectively). T1S positively correlated with CL in both groups (Pearson correlation coefficients of 0.588 in the NG and 0.504 in the DG). No significant difference was seen in TIA between the NG and DG.

**Conclusions:**

T1S was involved in the occurrence and development of cervical disc degeneration, and TIA could be considered as a constant morphological parameter in both the normal population and cervical disc degeneration patients.

**Electronic supplementary material:**

The online version of this article (10.1186/s12891-018-1951-8) contains supplementary material, which is available to authorized users.

## Background

The sagittal alignment of the spine is an important factor involved in deformity, degenerative diseases, surgical planning, and postoperative recovery [1, 2]. Much research has focused on lumbosacral alignment, where the pelvic incidence (PI), a constant morphological parameter, plays a significant role. The influence of pelvic incidence on lumbar lordosis and thoracic kyphosis has been widely accepted [3, 4].

Recent studies have demonstrated the importance of cervical sagittal alignment, because neck pain and functional disability could be caused by loss of cervical lordosis, besides trauma, tumour, disc degeneration, soft tissue inflammation, etc. [5]. Further studies have showed that cervical sagittal alignment is not influenced solely by cervical lordosis. A close correlation exists between cervical lordosis and cervical sagittal parameters such as T1 slope (T1S), neck tilt (NT), and thoracic inlet angle (TIA) [6]. Some investigators have described TIA as the cervical spine equivalent of PI [7]. To preserve a physiological NT of around 44°, a large TIA led to both an increased T1 slope and cervical lordosis, and vice versa [8]. Therefore, accurate analysis of cervical sagittal parameters is critical to the study of cervical sagittal alignment. Previous studies have measured these parameters on cervical radiographs of asymptomatic adults. However, as MRI have not been applied to distinguish the status of their cervical discs, some asymptomatic subjects with cervical disc degeneration or herniation that cannot be shown on cervical radiographs were most likely included. The prevalence of cervical disc degenerative changes in asymptomatic individuals has been reported as over 20% [9–11]. It suggests that previous studies of cervical sagittal alignment included subjects undiagnosed cervical disc degeneration or herniation, which makes the accurate data and correct relationship of cervical sagittal parameters of healthy cervical spine adults still unclear.

In the present study, all subjects underwent MRI to assess the condition of their cervical discs and select the healthy cervical spine adults. To our knowledge, this study is also the first study to compare data of cervical parameters on radiographs between the normal population and cervical disc degeneration patients. The purpose of the present study was to obtain accurate cervical sagittal parameters for both the healthy cervical spine adults and patients with cervical disc degeneration, and analyze the relationship and characteristics of the two groups.

## Methods

A retrospective analysis was performed of data from 50 normal control subjects whose cervical spine are healthy (normal group, NG) and 50 patients with cervical disc degeneration (degeneration group, DG), who visited the outpatient department or physical examination center of our hospital between January 2010 and September 2015. Each individual had both a cervical MRI and cervical radiographs.

The inclusion criteria of the NG were as follows: (1) enrolled asymptomatic volunteers whose age are between 20 and 50 years; (2) no history of diagnosis or treatment related to any part of the spine, all of them underwent both MRI and cervical radiographs; (3) no evidence of degenerative cervical discs on MRI, according to the modified Matsumoto’s classification [12] (Table 1); and (4) T1 vertebral body or upper end of sternum clearly visible on lateral radiograph (not obscured by the shoulder contour).

**Table 1:** Grading system of the modified Matsumoto’s classification

Decrease in signal intensity of intervertebral disc
0: As bright as or slightly less bright than healthy disc
1: Markedly darker than healthy disc
2: No signal
Posterior disc protrusion
0: No protrusion
1: Disc material protruding beyond the posterior margin of the vertebral body without cord compression
(less than one-third of dural sac)
2: Beyond vertebral body with cord compression (more than one-third of dural sac)
Disc space narrowing
0: 100–75% of height of upper healthy disc
1: 75–50% of height of upper healthy disc
2: Less than 50% of height of upper healthy disc

The subjects of the DG were selected by image data system in our hospital. We searched and selected subjects who had both a cervical MRI and cervical radiographs. The inclusion criteria of the DG were as follows: (1) age between 20 and 50 years; (2) no history of spinal surgery, trauma, deformities, or tumors; (3) symptomatic adults with degenerative cervical discs (≥1and ≤3) assessed via cervical MRI, according to the modified Matsumoto’s classification (Table 1); and (4) T1 vertebral body or upper end of sternum clearly visible on lateral radiograph (not obscured by the shoulder contour).

The cervical intervertebral discs, including C2/3 to C7/T1, were evaluated. Each disc was assessed using MRI by two independent spine surgeons for: (1) decrease in signal intensity of intervertebral disc; (2) posterior disc protrusion; and (3) disc space narrowing, according to the modified Matsumoto’s classification. Each reader evaluated twice at different time points. The final MRI result depended on their comprehensive opinions. A cervical disc was defined as normal if it is no more than grade 1 in all of the section of the modified Matsumoto’s classification, and degenerative if it was more than grade 1 in any section of the modified Matsumoto’s classification.

A standing lateral radiograph of the cervical spine was obtained with the subject in a neutral standing position, looking straight ahead. The parameters, including T1 slope, TIA, neck tilt, C2–C7 lordosis, sagittal vertical axis (SVA) C2–C7, cervical tilt, and cranial tilt were measured on the cervical radiograph.

### Cervical parameters

**Fig. 1:**
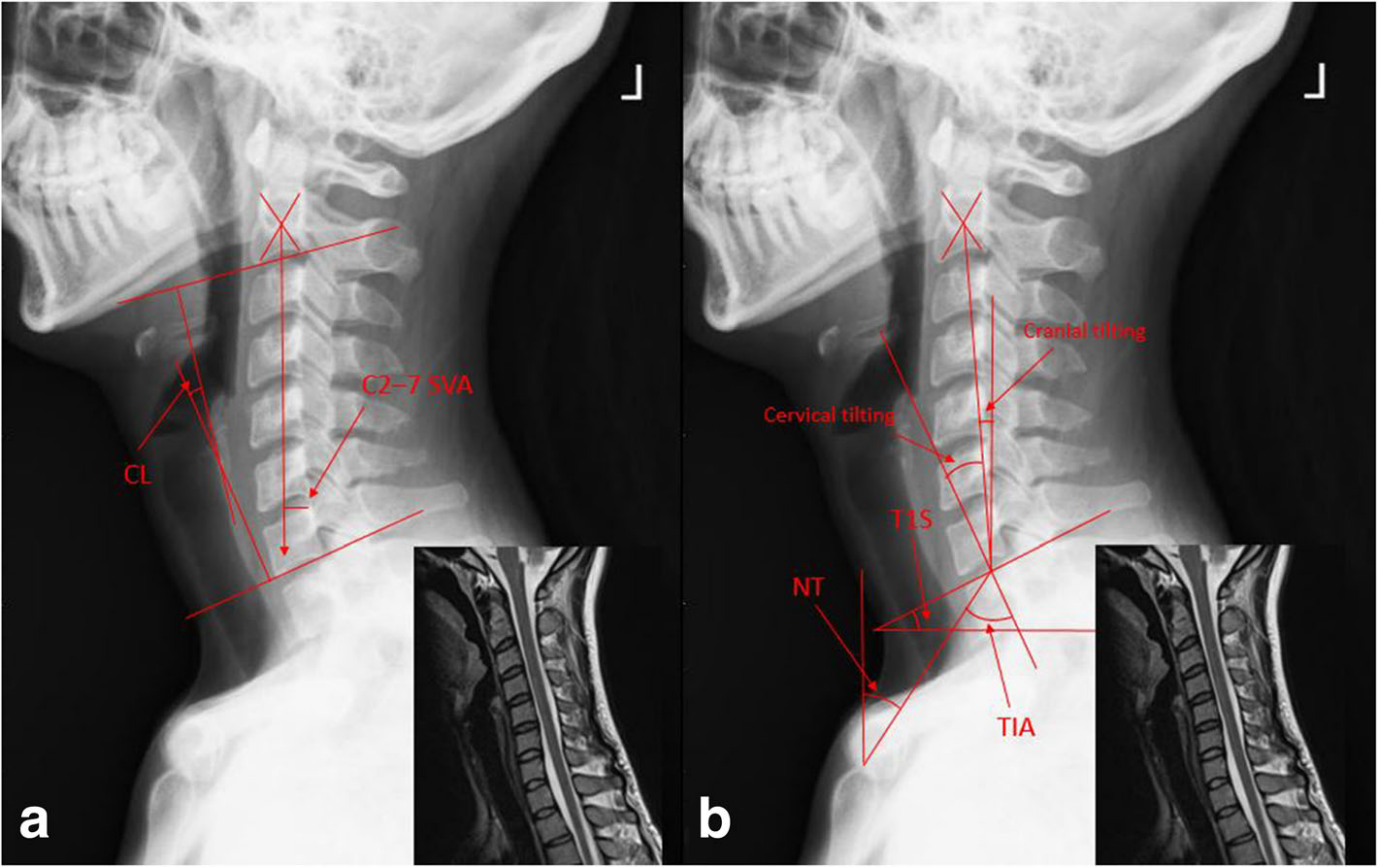
Measurements of radiological parameters. **a**: C2–7 lordosis (CL), C2–7 sagittal vertical axis (C2–7 SVA). **b**: T1 slope (T1S), thoracic inlet angle (TIA), neck tilt (NT), cervical tilting, cranial tilting

C2–7 lordosis (CL): the Cobb angle between the lower endplates of C2 and C7 (Fig. 1a).

C2–7 sagittal vertical axis (C2–7 SVA): the distance from the posterosuperior corner of C7 to a vertical line from the center of the C2 vertebra (Fig. 1a).

T1 slope (T1S): the angle between the upper endplate of T1 (T1UEP) and the horizontal (Fig. 1b).

Thoracic inlet angle (TIA): an angle formed by a vertical line from the center of the T1UEP, and a line connecting the center of the T1UEP and the upper end of the sternum (Fig. 1b).

Neck tilt (NT): an angle formed by a vertical line from the sternum tip and a line connecting the center of the T1UEP and the upper end of the sternum (Fig. 1b).

Cervical tilting: an angle formed between the vertical line from the center of T1UEP and the line from the center of T1UEP to the center of C2 vertebra (Fig. 1b).

Cranial tilting: an angle formed between the line from the center of the T1UEP to the dens and the SVA from the T1UEP (Fig. 1b).

T1S minus C2–7 lordosis (T1S-CL): the T1S angle minus the C2–7 lordosis.

Geometrically, TIA = T1S + NT while T1S = cervical tilting + cranial tilting.

### Statistical analysis

All measurements were determined using the picture archiving and communication system in the hospital by two independent spine surgeons and their average value adopted. Quantitative data were analyzed using t test, and categorical data were analyzed using the chi-squared test. The correlations between parameters of the normal and degenerative groups were analyzed using the Pearson correlation coefficient. Statistical analyses were performed using SPSS 22.0 (SPSS Inc., Chicago, IL, USA) and *P* < 0.05 was considered significant.

## Results

Comparison of the NG and DG (Additional files 1 and 2) yielded no significant differences in age, gender, C2–7 SVA, CL, TIA, cervical tilting, cranial tilting, or T1S-CL (Table 2). However, the mean T1S in the NG was 25.792 ± 5.13, significantly greater than in the DG, while the mean NT (44.6 ± 6.2) and the C2–7 SVA (12.0 ± 9.6) were significantly lower (Table 2).

**Table 2:** Data of normal group and degeneration group

	Normal group	Degeneration group	*P* value
No. of subjects (n)	50	50	
Age (years)	32.14 ± 9.19	35.02 ± 5.46	0.060
Gender (n)			
Male	25	30	0.315
Female	25	20	
C2–7 SVA (mm)	12.0 ± 9.6	16.7 ± 11.9	0.032*
CL (°)	19.2 ± 11.9	15.1 ± 12.2	0.093
TIA (°)	70.22 ± 6.8	71.5 ± 8.0	0.387
T1S (°)	25.8 ± 5.1	22.9 ± 7.0	0.020*
NT (°)	44.6 ± 6.2	48.6 ± 6.8	0.002*
Cervical tilt (°)	19.4 ± 6.9	16.7 ± 7.6	0.071
Cranial tilt (°)	5.4 ± 5.7	6.2 ± 6.5	0.824
T1S-CL (°)	6.6 ± 9.89	7.8 ± 10.5	0.569

*Indicates significant difference

There were significant relationships between C2–7 SVA and C2–7 lordosis, C2–7 SVA and T1S, C2–7 SVA and Cervical tilt,C2–7 SVA and Cranial tilt,C2–7 SVA and T1S-C2–7 lordosis, TIA and T1S, C2–7 lordosis and TIA, C2–7 lordosis and T1S, C2–7 lordosis and Cervical tilt, C2–7 lordosis and T1S-C2–7 lordosis, TIA and NT, TIA and T1S, TIA and Cervical tilt, T1S and NT, T1S and Cervical tilt, Cervical tilt and Cranial tilt, Cervical tilt and T1S-C2–7 lordosis, Cranial tilt T1S-C2–7 lordosis in both the NG and DG. (Tables 3 and 4).

**Table 3:** Pearson correlation of the parameters in the normal group

	C2–7 SVA	CL	TIA	T1S	NT	Cervical tilt	Cranial tilt	T1S-CL
Age	−0.345^a^	0.411^b^	0.268	0.194	0.128	0.320^a^	− 0.213	−0.397^b^
C2–7 SVA		−0.482^b^	− 0.074	0.310^a^	− 0.084	−0.638^b^	0.785^b^	0.592^b^
CL			0.303^a^	0.588^b^	− 0.166	0.600^b^	− 0.194	−0.906^b^
TIA				0.475^b^	0.681^b^	0.346^a^	0.010	−0.119
T1S					−0.320^a^	0.588^b^	0.193	−0.191
NT						−0.120	−0.145	0.035
Cervical tilt							−0.680^b^	− 0.420^b^
Cranial tilt								0.337^a^

^a^Significant correlation at the 0.05 level (2 tailed)

^b^Significant correlation at the 0.01 level (2 tailed)

**Table 4:** Pearson correlation of the parameters in the degeneration group

	C2–7 SVA	CL	TIA	T1S	NT	Cervical tilt	Cranial tilt	T1S-CL
Age	0.053	0.210	−0.003	0.063	−0.070	0.038	0.024	−0.200
C2–7 SVA		−0.475^b^	0.021	0.320^a^	− 0.144	−0.601^b^	0.875^b^	0.654^b^
CL			0.432^b^	0.504^b^	− 0.018	0.646^b^	− 0.212	−0.817^b^
TIA				0.610^b^	0.546^b^	0.434^b^	0.151	−0.091
T1S					−0.330^a^	0.607^b^	0.369^b^	0.087
NT						−0.124	− 0.211	−0.200
Cervical tilt							−0.515^b^	− 0.340^a^
Cranial tilt								0.491^b^

^a^Significant correlation at the 0.05 level (2 tailed)

^b^Significant correlation at the 0.01 level (2 tailed)

**Fig. 2:**
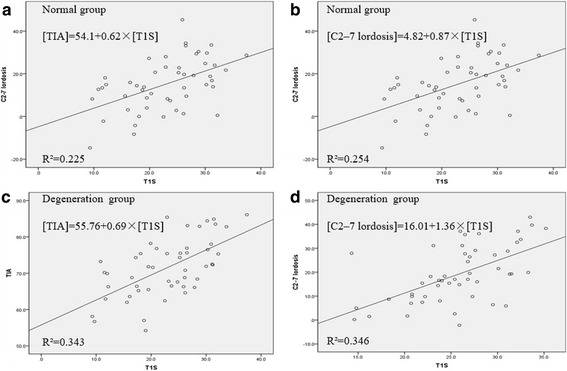
Linear regression analysis model of the parameters showed statistically significant relationships. **a**: T1 slope versus Thoracic inlet angle in NG; **b**: T1 slope versus C2–7 lordosis in NG; **c**: T1 slope versus Thoracic inlet angle in DG; **d**: T1 slope versus C2–7 lordosis in DG

T1 slope was the parameter that demonstrated significant correlations with most other parameters in both group, especially significantly correlated with TIA and C2–7 lordosis (Pearson correlation coefficients of 0.475 and 0.588 in the NG, 0.610 and 0.504 in the DG, Fig. 2).

## Discussion

The previous studies of spinal sagittal alignment focused on the thoracolumbar or spinopelvic region. Little attention has been paid to cervical sagittal alignment until recently. Several parameters were proposed to have a critical effect on cervical sagittal alignment. Some researchers consider TIA as a constant morphologic parameter, which significantly correlates with cervical sagittal alignment. T1 slope correlates highly with the other cervical parameters and plays an important role in the cervical sagittal alignment [7, 8]. Previous studies of cervical sagittal alignment were concentrated on asymptomatic adults; however, selection by radiographs without MRI may have resulted in enrolment of some asymptomatic patients with disc degeneration. X-ray usually only demonstrated relatively severe disc degeneration, while the early stage of degenerative disc and mild disc degeneration could not be found in radiographs. As a result, the high prevalence (usually over 20%) of the cervical disc degenerative change in the asymptomatic individuals were reported in previous studies [10–12]. Therefore, the cervical sagittal parameters of normal population and the impact of disc degeneration in cervical sagittal alignment are still uncertain.

In the present study, all individuals who visited the outpatient department or physical examination center of the hospital were screened, and they had both cervical MRI and cervical radiographs obtained together in the last five years, and selected asymptomatic adults with normal imaging. To the best of our knowledge, this is the first study to investigate the cervical parameters of cervical radiographs of the normal population screened via MRI, as well as, the first comparative study of cervical parameters data on radiographs between the normal population and cervical disc degeneration patients.

In the present study, the mean TIA of the NG and DG were 70.22 ± 6.8 and 71.5 ± 8.0, respectively, similar to data in previous studies [4, 8]. There was no significant differences between the TIA in the two groups, indicating that TIA was not involved in the development of cervical disc degeneration, which could be considered a constant parameter of approximately 70°. The relationships between TIA, T1S, and NT in both groups were similar to previous studies. There were positive correlations between TIA and T1S, and TIA and NT.

Before the study, we suspected that the result of normal population might be different from previous studies of asymptomatic subjects since we excluded the disc degeneration asymptomatic subjects. However, most parameters including T1S (25.8 ± 5.1) in the NG was similar to asymptomatic subjects in previous studies, [13] even though asymptomatic subjects in previous studies might have contained some subjects with disc degeneration. We considered that the number of degenerative cervical discs generally was small and the level of degeneration was mild while the subjects with cervical disc degeneration were asymptomatic; therefore, we infer that a small number of degenerative discs and mild disc degeneration might not cause an obvious impact on T1S.

Since subjects in DG were selected with at least one degenerative disc, usually relatively severe degeneration in our study, the degeneration level of disc in DG was obviously more serious than the asymptomatic subjects with disc degeneration in previous studies, there were significant difference between some parameters in the NG and DG. C2–7 SVA and T1S in the DG were significantly lower than in the NG (16.7 ± 11.9 vs 12.0 ± 9.6, and 22.9 ± 7.0 vs 25.8 ± 5.1, respectively) and NT in the DG was significantly greater than in the NG (48.6 ± 6.8 vs 44.6 ± 6.2). We speculated a possible pathological process of cervical disc degeneration according to our findings. As the T1S positively correlated to CL (Pearson correlation coefficients of 0.588 in the NG and 0.504 in the DG), lower T1S decreased the CL to obtain a sagittally balanced cervical spine, and led to a relatively straight cervical spine. This mechanism might increase the stress due to gravity and accelerate cervical disc degeneration, which could explain why T1S in the DG was lower than in the NG (22.9 ± 7.0 vs25.8 ± 5.1). However, as the effect of T1S on cervical disc degeneration, degenerative discs might also have an impact on T1S and other cervical parameters which could be gradually changed due to the development of disc degeneration. Although we could define that lower T1S was a risk of the occurrence or development of cervical disc degeneration according to our finding that significantly different T1S in the normal population and cervical disc degeneration patients, we could not determine the causal relationship between T1S and cervical disc degeneration without a longitudinal study, they might interact with each other. However, this study could provide a new finding that some cervical parameters including T1S were significantly different in normal population and degeneration patients, and also a new research direction of the relationship between cervical alignment and cervical disc degeneration that we need more researches especially longitudinal studies to determine.

Cervical sagittal balance was important and had regulatory mechanism. We speculated a possible compensatory principle according to our result: high T1 slope in either group increased the CL, which correlated with a lower SVA C2–7 (Pearson correlation coefficients of − 0.482 in the NG and − 0.475 in the DG). We considered that T1S-CL was a better parameter than T1S to demonstrate the compensatory mechanism of the cervical spine, analogous to PI-LL in the lumbopelvic spine [14, 15]. In our study, T1S-CL significantly correlated with C2–7 SVA (Pearson correlation coefficients of 0.592 in the NG and 0.654 in the DG). A large T1S-CL increased the C2–7 SVA and an excessively large C2–7 SVA that caused cervical spine decompensation would contribute to imbalance in the cervical spine. In addition, as some researchers found that T1S-CL significantly correlated with postoperative neck disability index scores in posterior cervical fusion surgery, [16, 17] future studies could estimate the value of T1S-CL in predicting and preventing cervical axial symptoms.

However, there are several limitations in this study. Firstly, the relatively small sample size prevented division of the DG into subgroups according to the amount and level of disc degeneration. Secondly, there was a relatively narrow age distribution; further studies should enroll normal volunteers with a wider distribution. Finally, this is a cross-sectional comparative study that lacked longitudinal data to demonstrate an association between T1S and the occurrence and development of cervical disc degeneration.

## Conclusion

The findings indicate that lower T1S might be a risk of the occurrence and development of cervical disc degeneration and provide a new thought about the relationship between T1S and cervical disc degeneration. Our findings also demonstrate that TIA could be considered as a constant morphological parameter in the normal population and in cervical disc degeneration patients.

## Additional files

Additional file 1: Original data-NG group. The Parameters data in the Normal Group. (XLSX 13 kb)

Additional file 2: Original data-DG group. The Parameters data in the Degeneration Group. (XLSX 13 kb)

## References

[1] Vialle R, Levassor N, Rillardon L, Templier A, Skalli W, Guigui P (2005). Radiographic analysis of the sagittal alignment and balance of the spine in asymptomatic subjects. J Bone Joint Surg.

[2] Schuller S, Charles YP, Steib JP (2011). Sagittal spinopelvic alignment and body mass index in patients with degenerative spondylolisthesis. Eur Spine J.

[3] Boulay C, Tardieu C, Hecquet J, Benaim C, Mouilleseaux B, Marty C (2006). Sagittal alignment of spine and pelvis regulated by pelvic incidence: standard values and prediction of lordosis. Eur Spine J.

[4] Jang JS, Lee SH, Min JH, Kim SK, Han KM, Maeng DH (2007). Surgical treatment of failed back surgery syndrome due to sagittal imbalance. Spine.

[5] Tang JA, Scheer JK, Smith JS, Deviren V, Bess S, Hart RA (2012). The impact of standing regional cervical sagittal alignment on outcomes in posterior cervical fusion surgery. Neurosurgery.

[6] Knott PT, Mardjetko SM, Techy F (2010). The use of the T1 sagittal angle in predicting overall sagittal balance of the spine. Spine J.

[7] Lee SH, Son ES, Seo EM, Suk KS, Kim KT (2015). Factors determining cervical spine sagittal balance in asymptomatic adults: correlation with spinopelvic balance and thoracic inlet alignment. Spine J.

[8] Lee SH, Kim KT, Seo EM, Suk KS, Kwack YH, Son ES (2012). The influence of thoracic inlet alignment on the craniocervical sagittal balance in asymptomatic adults. J Spinal Disord Tech.

[9] Teresi LM, Lufkin RB, Reicher MA, Moffit BJ, Vinuela FV, Wilson GM (1987). Asymptomatic degenerative disk disease and spondylosis of the cervical spine: MR imaging. Radiology.

[10] Lao LF, Zhong GB, Li QY, Liu ZD (2014). Kinetic magnetic resonance imaging analysis of spinal degeneration: a systematic review. Orthop Surg.

[11] Matsumoto M, Fujimura Y, Suzuki N, Nishi Y, Nakamura M, Yabe Y (1998). MRI of cervical intervertebral discs in asymptomatic subjects. J Bone Joint Surg Br.

[12] Matsumoto M, Okada E, Toyama Y, Fujiwara H, Momoshima S, Takahata T (2013). Tandem age-related lumbar and cervical intervertebral disc changes in asymptomatic subjects. Eur Spine J.

[13] Janusz P, Tyrakowski M, Glowka P, Offoha R, Siemionow K (2015). Influence of cervical spine position on the radiographic parameters of the thoracic inlet alignment. Eur Spine J.

[14] Kim YJ, Bridwell KH, Lenke LG, Cheh G, Baldus C (2007). Results of lumbar pedicle subtraction osteotomies for fixed sagittal imbalance: a minimum 5-year follow-up study. Spine.

[15] Lafage V, Ames C, Schwab F, Klineberg E, Akbarnia B, Smith J (2012). Changes in thoracic kyphosis negatively impact sagittal alignment after lumbar pedicle subtraction osteotomy: a comprehensive radiographic analysis. Spine.

[16] Iyer S, Nemani VM, Nguyen J, Elysee J, Burapachaisri A, Ames CP (2016). Impact of cervical sagittal alignment parameters on neck disability. Spine.

[17] Hyun SJ, Kim KJ, Jahng TA, Kim HJ (2016). Relationship between T1 slope and cervical alignment following multilevel posterior cervical fusion surgery: impact of T1 slope minus cervical lordosis. Spine.

